# Increased Serum PAI-1 Levels in Subjects with Metabolic Syndrome and Long-Term Adverse Mental Symptoms: A Population-Based Study

**DOI:** 10.1155/2010/501349

**Published:** 2010-03-14

**Authors:** Anne Huotari, Soili M. Lehto, Leo Niskanen, Karl-Heinz Herzig, Jukka Hintikka, Heli Koivumaa-Honkanen, Tommi Tolmunen, Kirsi Honkalampi, Noora Kaikkonen, Heimo Viinamäki

**Affiliations:** ^1^Division of Physiology, Institute of Biomedicine, Biocenter Oulu, University of Oulu, 90014 Oulu, Finland; ^2^Department of Psychiatry, Institute of Clinical Medicine, Kuopio University Hospital, University of Eastern Finland, 70210 Kuopio, Finland; ^3^Kuopio Psychiatric Center, P. O. Box 1777, 70211 Kuopio, Finland; ^4^Department of Medicine, Institute of Clinical Medicine, Kuopio University Hospital, University of Eastern Finland, 70210 Kuopio, Finland; ^5^Department of Psychiatry, University of Oulu & Lapland Hospital District, 97140 Rovaniemi, Finland

## Abstract

Depression is an independent risk factor for cardiovascular diseases and is associated with metabolic syndrome (MetS). Levels of plasminogen activator inhibitor-1 (PAI-1), an inhibitor of tissue-type and urokinase-type plasminogen activators, are associated with MetS. To clarify the role of PAI-1 in subjects with long-term adverse mental symptomatology (LMS; including depression) and MetS, we measured circulating PAI-1 levels in controls (*n* = 111), in subjects with MetS and free of mental symptoms (*n* = 42), and in subjects with both MetS and long-term mental symptoms (*n* = 70). PAI-1 increased linearly across the three groups in men. In logistic regression analysis, men with PAI-1 levels above the median had a 3.4-fold increased likelihood of suffering from the comorbidity of long-term adverse mental symptoms and MetS, while no such associations were detected in women. In conclusion, our results suggest that in men high PAI-1 levels are independently associated with long-term mental symptomatology.

## 1. Introduction

An increased prevalence of metabolic syndrome (MetS) is a common finding among depressed patients [1]. Furthermore, depression is an independent risk factor for the development of cardiovascular diseases [2]. Nevertheless, causal relationships between these factors remain unclear [3], although several behavioral or physiological mechanisms have been suggested [4]. According to previous studies, increased serum plasminogen activator inhibitor-1 (PAI-1) levels are associated with metabolic changes [5], and possibly also with mental health problems [6]. Furthermore, depression is a significant public health care issue worldwide, and the incidence of metabolic syndrome is increasing drastically. The relationship between these two conditions is bidirectional [7]. Nevertheless, the factors regulating these associations are unclear.

Plasminogen activator inhibitor-1 (PAI-1) is a major physiological inhibitor of tissue-type (tPA) and urokinase-type plasminogen (uPA) activators. It also possesses several other roles in human physiology. PAI-1 belongs to the family of serine protease inhibitors (SERPINs), and it is an inhibitor of intravascular fibrinolysis and cell-associated proteolysis. Under normal physiological conditions, PAI-1 is synthesized by the liver, smooth muscle cells, adipocytes and platelets [8]. However, in pathological conditions, such as atherosclerosis, endothelial cells and other inflammatory-stimulated cells secrete notable amounts of PAI-1 [8]. In addition, abdominal obesity is related to elevated PAI-1 plasma concentrations, and thus increased amounts of adipose tissue might contribute to PAI-1 secretion, especially in obese subjects [9]. Circulating PAI-1 is found in two conformationally distinct forms: an active form, which has a relatively short plasma half-life (~30 min), and a latent inactive form [10]. Binding to a carrier protein, vitronectin, stabilizes the active form of PAI-1 [11]. PAI-1 is also found in cerebrospinal fluid [12]. tPA can cross the blood-brain barrier (BBB) [13], and is synthesized in several brain regions [14]. Furthermore, Yepes et al. reported that tPA increased BBB permeability after ischemic stroke [15]. Whether PAI-1 itself is capable of crossing the BBB is currently unknown. However, Hino et al. showed immunohistochemically the localization of type 1 plasminogen activator inhibitor in human brain tissues [16]. 

Recent findings suggest a link between elevated PAI-1 levels and major depressive disorder (MDD) [17, 18]. However, the role of metabolic syndrome was not assessed in these studies. As PAI-1 is an independent and true component of metabolic syndrome, we examined the additional effect of mental symptoms on the previously observed association between metabolic syndrome and PAI-1. Moreover, the possible gender differences have not been addressed in previous studies. Thus, in the present study we examined the role of long-term adverse mental symptoms (LMS) in subjects with metabolic syndrome in the regulation of PAI-1 levels in a population-based sample of both men and women.

## 2. Methods

### 2.1. Study Population

The present study was a part of the longitudinal population-based Kuopio Depression Study (KUDEP) in the central-eastern part of Finland [1, 19, 20]. The random general population sample (*n* = 3004) was initially selected from the National Population Register in 1998. Study questionnaires were mailed to the subjects. The baseline sample consisted of 2050 respondents aged 25–65 years. Follow-ups were performed in 1999 (*n* = 1722) and in 2001 (*n* = 1593). Altogether, 1347 subjects responded three times. 

The sample of the present study was from subgroups of this 3-year followup study. The inclusion criteria for the sample were based on the presence or absence of self-reported mental symptoms prevailing at baseline and at both follow-ups. First, we selected subjects who reported at least one of the following at each followup: high Beck Depression Inventory scores (BDI-21 score >9) [21], alexithymic features according to the Toronto Alexithymia Scale (TAS-20 score >58) [22], or life dissatisfaction with the four-item Life Satisfaction Scale (LS score >11) [23]. The mean BDI scores (SD) for these subjects with long-term mental symptoms (LMS group) were 14.7 (8.8) in 1998, 15.6 (10.0) in 1999, and 15.2 (9.8) in 2001, indicating clinically significant prevailing depressive symptoms during the followup. A control group of those not fulfilling any of these criteria at any measurement point was formed. Their respective BDI scores (SD) were 2.7 (2.7) in 1998, 2.9 (3.0) in 1999, and 2.4 (2.8) in 2001. There were no differences between these groups in age or gender distributions (data not shown).

Seven years from the baseline, during clinical assessment, the subjects in both sub-samples completed a questionnaire relating to their socio-demographic background. The questionnaire included the following variables: age, sex, and marital status (married or living with a partner versus living alone). The subjects were also asked about their smoking status (daily smoker: yes/no), frequency of alcohol consumption (more than twice per week versus less), previous physician-diagnosed illnesses, and whether they had taken any psychotropic medication during the preceding week. Data on the use of somatic medications during the study period were obtained from the register of the National Agency for Medicines and the Social Insurance Institute. Depressive symptoms were assessed with the BDI [21]. 

For this study focusing on the relationships between PAI-1, MetS, and mental symptomatology, the subjects were further divided into three groups: (1) controls (subjects without both MetS and LMS; 42 men and 69 women), (2) control + MetS (subjects with MetS but without LMS; 25 men and 17 women) and (3), LMS + MetS (subjects with comorbid MetS and LMS; 42 men and 28 women). The subjects with LMS but without MetS were omitted from the current study.

Approval for the study was obtained from the Ethics Committee of Kuopio University Hospital and the University of Kuopio, and the study protocol was in accordance with the latest version of the Declaration of Helsinki. All participants provided written informed consent before entering the study.

### 2.2. Measurements

The laboratory measurements were carried out in the laboratory of Kuopio University Hospital. The subjects came for venous blood sampling at 8 am, after having been instructed to fast for the previous 12 hours. Serum total cholesterol (TC), high-density lipoprotein cholesterol (HDL-C), triglyceride (TG), and fasting plasma glucose measurements were carried out according to routine protocols. Enzymatic methods (Thermo Electron Co, Finland) were used for all measurements (TC: Konelab CHOLESTEROL; HDL-C: Konelab HDL-CHOLESTEROL, TG: Konelab TRIGLYCERIDES; fasting plasma glucose: Konelab GLUCOSE). The total variations in the utilized methods were 1.6%, 3.7%, 4.8%, and 3.1%, respectively. The samples were analyzed using a Konelab 60i Clinical Chemistry Analyzer (Thermo Electron Co.). Waist circumference was measured at the midpoint between the lowest rib and the iliac crest. A research nurse measured the blood pressure after 10 minutes of rest in the sitting position at 2-minute intervals. Systolic and diastolic blood pressures were obtained as the mean of three measurements. Height and body weight were measured in light clothing without shoes, and the body mass index (BMI; kg/m^2^) was calculated. 

MetS was diagnosed according to the modified criteria of the National Cholesterol Education Program Adult Treatment Panel III [NCEP ATP III; 1,19,20] based on the presence of 3 or more of the following findings: fasting plasma glucose levels ≥5.6 mmol/L, serum triglycerides ≥1.7 mmol/L, serum high-density lipoprotein cholesterol (HDL-C) < 1.0 mmol/L in men and <1.3 mmol/L in women, systolic blood pressure ≥130 mmHg and/or diastolic blood pressure ≥85 mmHg, or waist girth >102 cm for men and >88 cm for women. 

For PAI-1 analyses, the venous blood samples were stored at −80°C until run. PAI-1 was analyzed with a human serum adipokine LincoPlex kit (Millipore, MA, USA) using a Bio-Plex Suspension Array System (Bio-Rad Laboratories Pty Ltd; Hercules, CA, USA). Before the analyses, samples were centrifuged for 15 minutes at 3000 rpm. For the analyses the samples were diluted in the appropriate sample matrix to 1 : 400 according to the kit instructions. The assays were performed according to the manufacturer's instructions. The intra-assay and interassay variations for analyses were <7.9% and 21%, respectively.

### 2.3. Statistical Analysis

Differences between the study groups were assessed using the chi-squared test for categorical variables and, when needed, exact *P*-values were calculated with Fisher's exact test. Due to the skewed distribution of the PAI-1 data, the non-parametric Kruskal-Wallis test was used for PAI-1 and one-way ANOVA for other continuous variables. For post-hoc testing of PAI-1, the differences between the groups were assessed with the Mann-Whitney *U*-test. The tests were performed separately for men and women.

Logistic regression analysis (method: enter) was performed to examine factors independently associated with the likelihood of belonging to the LMS + MetS group. The analyses were performed separately for men and women. The regression model was adjusted for age, marital status, alcohol consumption, daily smoking, cardiovascular disease other than hypertension, and overweight (BMI ≥ 25). The covariates were chosen based on the statistical significance between the groups (BMI, alcohol consumption, smoking, marital status) and the known influence on PAI-1 levels (BMI [5], cardiovascular disease [8] and age [24]). A median-based cut-off was used for PAI-1 levels in order to improve interpretability and optimize statistical power in the analyses. PAI-1 medians were calculated separately for men and women (men: median = 32.58 ng/mL, lower median half 0.00–32.13, upper median half 32.58–147.35; women: median 28.11 ng/mL, lower median half 0.00–28.05, upper median half 28.16–115.38). In addition, as a second step, we performed the same regression analysis using PAI-1 levels as a continuous variable. Other covariates remained identical to the first model, and belonging to the group of LMS + MetS was still the dependent variable. Two-tailed *P*-values <.05 were considered to indicate statistical significance in all analyses, which were performed using SPSS software version 14.0 for Windows.

## 3. Results

Characteristics of the study population are described in Table 1. BDI scores were highest in the LMS + MetS group and BMI was lowest in the control group for both women and men. In men, the highest number of current smokers was observed in the LMS + MetS group, whereas the number of frequent alcohol users was highest in the control group. In women, the subjects in the LMS + MetS group were most often living alone. The prevalence of cardiovascular disease was low in all the groups. 

**Figure 1:**
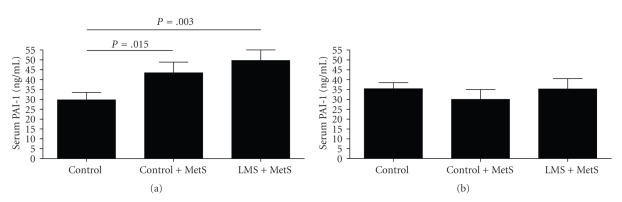
Serum PAI-1 levels in (a) men and (b) women. Data are expressed as means ± standard error of the mean. Statistical analysis was performed with the Kruskal-Wallis test and post hoc testing between individual groups with the Mann-Whitney *U*-test. Only statistically significant *P*-values are shown.

Men belonging to the control + MetS group had higher PAI-1 levels when compared with controls (mean values: 43.42 ng/mL (SEM 5.39) versus 29.76 ng/mL (SEM 3.72), *P =* .015; Figure 1(a)). Furthermore, the LMS + MetS group had elevated PAI-1 levels compared with controls (mean 49.68 ng/mL (SEM 5.28), versus 29.76 ng/mL (SEM 3.72), *P =* .003; Figure 1(a)). The mean increase in PAI-1 levels followed a linear trend across the three groups (control, control + MetS, LMS + MetS; *P =* .002). Interestingly, PAI-1 levels in women were similar in all groups (Table 1, Figure 1(b)). Men had higher mean PAI-1 levels than women, but the difference was not statistically significant (men: 40.57 ng/mL (SEM 2.88); women: 34.58 ng/mL (SEM 2.37), *P =* .236).

### 3.1. Effect of Medications

The consumption of lipid-lowering agents (mainly statins) did not differ between the groups for men or women (number (%) of male users: controls 15 (36%), control + MetS 9 (36%), and LMS + MetS 10 (24%), *P =* .42; number (%) of female users: controls 13 (19%), control + MetS 6 (35%), and LMS + MetS 8 (29%), *P =* .28). The consumption of lipid-lowering agents did not affect the mean total PAI-1 levels in men (men consuming lipid-lowering drugs 40.05 ng/mL (SEM 4.84) versus not consuming 40.81 ng/mL (SEM 3.59); *P =* .90), or in women (34.64 ng/mL (5.22) versus 34.56 ng/mL (SEM 2.66), resp.; *P =* .99). The self-reported consumption of antidepressants was similar in all groups for men (controls: *n* = 4 (10%); control + MetS: *n* = 3 (12%); LMS + MetS: *n* = 8 (19%); *P =* .43) and highest in control + MetS and LMS + MetS groups for women (controls: *n* = 3 (4%); control + MetS: *n* = 5 (29%); LMS + MetS: *n* = 7 (25%); *P =* .002). Those men using antidepressants had slightly lower PAI-1 levels than the other men, but the difference was not statistically significant (37.00 ng/mL (SEM 7.26) versus 41.14 ng/mL (SEM 3.15), resp.; *P =* .62). The opposite finding was recorded in women, and there was a trend towards statistical significance (45.33 ng/mL (SEM 9.56), versus 32.95 ng/mL (SEM 2.29), resp.; *P =* .08).

There were no significant differences in the number of women receiving sex hormones in the three study groups (controls: *n* = 20 (29.0%); control + MetS: *n* = 7 (41.2%); LMS + MetS: *n* = 7 (25.0%); *P =* .501). Furthermore, the difference in mean PAI-1 levels between the women with (*n* = 34) or without (*n* = 80) the use of sex hormones did not reach statistical significance (PAI-1: 30.41 ng/mL (SEM 3.61) versus PAI-1: 36.35 ng/mL (SEM 2.99), resp.; *P =* .253).

### 3.2. Multivariate Analyses

In logistic regression analysis, men having PAI-1 levels above the median had 3.4-fold higher risk for belonging to the LMS + MetS group (OR 3.40, 95% CI 1.23–9.40, *P =* .018) (Table 2), independently of potential confounders. In addition, smoking and overweight were independently associated with the likelihood of belonging to the LMS + MetS group for men (OR 9.93, 95% CI 2.56–38.57; OR 3.85, 95% CI 1.04–14.21, resp.). However, PAI-1 levels above the median did not predict mental symptomatology in women (Table 2). In men, smoking and overweight were independent predictors of MetS and LMS (OR 9.93, 95% CI 2.56–38.57, *P =* .001 and OR 3.85, 95% CI 1.04–14.21, *P =* .043, resp.). These factors were also significant predictors in women (smoking OR 7.86, 95% CI 1.55–39.73, *P =* .013, and overweight OR 27.36, 95% CI 3.17–235.77 and *P =* .003), and in women marital status was also significant (single OR 5.22, 95% CI 1.16–23.55, *P =* .032). As a second step, we used PAI-1 levels as a continuous variable to assess the risk of long-term mental symptoms and MetS. The results were essentially the same in men, as we observed that each one unit increase in PAI-1 levels increased the likelihood of belonging to LMS + MetS group by 2.3% (OR 1.023, 95% CI 1.01–1.04, *P =* .008) in men. Furthermore, smoking (OR 9.07, 95% CI 2.40–34.24, *P =* .001) and overweight (OR 4.33, 95% CI 1.17–16.00, *P =* .028) remained significantly associated with belonging to the LMS + MetS group in men. In women, the same predictors were significant as in the first model utilizing median cut-off of PAI-1 (smoking OR 7.47, 95% CI 1.50–37.30, *P =* .014, overweight OR 28.17, 95% CI 3.25–243.78, *P =* .02 and marital status single OR 4.95, 95% CI 1.09–22.45, *P =* .038).

## 4. Discussion

To the best of our knowledge, this is the first study to demonstrate the cumulative effect of long-term adverse mental symptoms and metabolic syndrome on serum PAI-1 levels in men. However, no such differences could be observed in women. 

Elevated PAI-1 levels have been linked with depression [17, 18, 25]. In addition, associations with vital exhaustion [26–28] and anxiety with agoraphobia or social phobia [29] have been reported. Furthermore, a single nucleotide polymorphism (SNP) found in the PAI-1 gene was linked to MDD and the antidepressant response [30]. The link between depression and PAI-1 levels could be explained by the inhibitory effect of PAI-1 on tPA. tPA is the main thrombolytic enzyme in blood, and it also promotes pathways associated with synaptic plasticity and cell death in the brain [15]. In addition, after ischemic stroke it also increases BBB permeability [15]. Brain-derived neurotrophic factor (BDNF), a member of the neurotrophin family that regulates neuronal plasticity and survival, might explain the connection between depression and the tPA system [31–33].

Direct evidence for PAI-1 crossing the blood-brain barrier has not currently been established, but PAI-1 is found in certain brain regions and in the cerebrospinal fluid [12, 16]. In patients with multiple sclerosis there is a negative correlation between PAI-1 levels in CSF and serum, which might suggest that there is an active transport mechanism through the BBB [34]. Whether PAI-1 directly causes the psychiatric symptoms in the central nervous system or whether the actions are indirect or mediated via the modulation of the BBB by tPA system is currently unknown.

Most of the earlier reports on the association between depression and the tPA system have been exclusively conducted on male subjects. Exceptions include the studies of Eskandari et al. [17] and Matthews et al. [25] on young premenopausal and perimenopausal women, respectively, which yielded similar findings to those for men in other studies. Hence, the gender difference found in the current study is novel and warrants further investigation.

The use of simvastatin may reduce the expression of the PAI-1 gene, and thereby reduce PAI-1 activity [35]. Moreover, other statins may have similar effects [36]. Thus, it has been suggested that statins might actually protect against depression [37]. In our study population, total cholesterol levels were similar in both genders. Furthermore, no differences were observed in the use of statins between the groups for either gender, and the PAI-1 levels were similar in subjects with or without the use of lipid-lowering drugs, mainly statins.

Increased circulating PAI-1 is strongly linked to MetS and is an independent component of the syndrome [38]. In our sample, men with MetS (control + MetS) and LMS + MetS had significantly higher PAI-1 levels than controls, but this difference was not seen in women. The findings in the female sample require confirmation in larger cohorts. However, in support of our findings, the correlations between cardiovascular disease traits and circulating PAI-1 levels have been shown to be greater in men than in women [39]. Postmenopausal women not receiving estrogen replacement have been reported to have higher PAI-1 levels compared to those on hormone replacement therapy [40]. Nevertheless, although we were unable to specifically examine the effect of the menopausal status in the female sample, we observed no differences in the levels of PAI-1 between women with or without sex hormones. 

As a limitation of the study, we measured total circulating PAI-1, not only the active form. However, in a recent investigation in a similar assay to ours from the same vendor, total and active PAI-1 correlated [41]. Thus, it could be considered that the measurement of total PAI-1 reflects the amount of active PAI-1. Nevertheless, a weakness of the current study setting is the low number of subjects in the study groups, which reduced the statistical power of our analysis. Moreover, we used self-report questionnaires instead of diagnostic criteria to evaluate adverse mental symptoms. Utilizing diagnostic criteria might have provided better comparability with other studies on the depression-related biological alterations. Nevertheless, in line with our study, dimensional rather than categorical (i.e., diagnosis-based) evaluation of depression has been suggested [42]. In addition to the PAI-1 assay, measurement of serum tPA could have provided a more comprehensive assessment of the prothrombotic status of the subjects [43].

In conclusion, high PAI-1 levels were associated with long-term adverse mental symptoms in men, regardless of the adjustment for potential confounders. No such association was detected in women. Hence, increased PAI-1 levels might partly explain the association between MetS and depression, as well as the increased risk for cardiovascular illnesses in depressed subjects. 

## Figures and Tables

**Table 1:** Group characteristics. Values are expressed as counts (%), except age, total cholesterol, BMI, BDI, and PAI-1, which are expressed as means (SEM).

	Men				Women			
	Control (*n* = 42)	Control + MetS (*n* = 25)	LMS + MetS (*n* = 42)	*P-*value*	Control (*n* = 69)	Control + MetS (*n* = 17)	LMS + MetS (*n* = 28)	*P-*value*

Age (years)	58.24 (1.61)	55.80 (2.19)	56.67 (1.35)	.592	55.59 (1.17)	60.82 (1.79)	55.18 (1.84)	.099
Marital status (single) *n* (%)	4 (10)	3 (12)	8 (19)	.430	5 (7)	1 (6)	9 (32)	.004
Total holesterol (mmol/L)	4.92 (0.14)	4.96 (0.17)	5.02 (0.98)	.891	5.19 (0.11)	5.17 (0.98)	5.03 (0.09)	.750
BMI (kg/m^2^)	24.82 (0.36)	29.56 (0.72)	29.79 (0.74)	<.001	25.98 (0.56)	31.78 (1.41)	32.37 (0.93)	<.001
Alcohol consumption (more than 2 × week) *n* (%)	13 (31)	1 (4)	10 (24)	.034	6 (9)	4 (24)	3 (11)	.258
Current smokers *n* (%)	5 (12)	1 (4)	13 (31)	.016	6 (9)	1 (6)	8 (29)	.098
Cardiovascular disease *n* (%)	0 (0)	2 (8)	2 (5)	.223	5 (7)	1 (6)	3 (11)	.358
Lipid-lowering drugs *n* (%)	15 (36)	9 (36)	10 (24)	.420	13 (19)	6 (35)	8 (29)	.282
BDI score 2005	2.73 (0.42)	3.12 (0.56)	12.76 (1.48)	<.001	3.86 (0.53)	2.59 (0.61)	13.54 (1.51)	<.001
PAI-1 (ng/mL)	29.76 (3.72)	43.42 (5.39)	49.68 (5.28)	.008	35.40 (3.07)	30.00 (5.01)	35.34 (5.21)	.724

*P values calculated with one-way ANOVA for age, total cholesterol, BMI, and the BDI score, with the Kruskal-Wallis test for PAI-1 and with the *χ* ^2^ test for alcohol consumption, smoking, marital status, cardiovascular disease, and the consumption of lipid-lowering drugs.

BDI: Beck Depression Inventory

BMI: Body mass index

SEM: Standard error of the mean.

**Table 2:** Odds ratios (OR) with 95% confidence intervals for the risk of the long-term mental symptoms and metabolic syndrome (LMS + MetS) in men and women.

	Men			Women		
	OR	95% CI	*P*-value	OR	95% CI	*P*-value

PAI-1 > median	3.40	1.23–9.40	.018	1.018	0.339–3.05	.975
Smoking	9.93	2.56–38.57	.001	7.86	1.55–39.73	.013
Overweight	3.85	1.04–14.21	.043	27.36	3.17–235.77	.003
Cardiovascular disease	1.72	0.17–17.57	.646	1.82	0.33–10.04	.492
Age	1.02	0.97–1.07	.373	0.97	0.91–1.04	.408
Marital status (single)	2.57	0.69–9.59	.160	5.22	1.16–23.55	.032
Alcohol more than 2 × week	1.07	0.37–3.08	.904	0.65	0.12–3.53	.617
